# Comparative effectiveness and safety of topiramate versus amitriptyline for the prophylactic management of chronic migraine: a prospective comparative study

**DOI:** 10.3389/fpain.2026.1923623

**Published:** 2026-09-24

**Authors:** Mohammed Matoog Karami, Amber Hassan, Amal Alkhotani, Mazen Basheikh, Adnan Badahdah, Raed Gasemaltayeb, Ahmed Bamaga, Taghreed Alsinani, Rakan Bokhari, Abdulrahman Almazrooa, Siti Sarah Maidin, Humaira Waseem, Muhammad Anjum, Maher Kurdi

**Affiliations:** 1 Department of Clinical Physiology, Faculty of Medicine, King Abdulaziz University, Jeddah, Saudi Arabia; 2 Department of Neurogenetics, Istituto Neurologico Nazionale a Carattere Scientifico, IRCCS, Pavia, Italy; 3 Department of Medicine, College of Medicine, Umm Al-Qura University, Jeddah, Saudi Arabia; 4 Department of Internal Medicine, Faculty of Medicine, University of Jeddah, Jeddah, Saudi Arabia; 5 Department of Internal Medicine, Faculty of Medicine, King Abdulaziz University, Rabigh, Saudi Arabia; 6 Department of Anaesthesia and Critical Care, Faculty of Medicine, King Abdulaziz University, Jeddah, Saudi Arabia; 7 Department of Neurosurgery, King Fahad General Hospital, Jeddah, Saudi Arabia; 8 Department of Surgery, Faculty of Medicine, King Abdulaziz University, Jeddah, Saudi Arabia; 9 Department of Anaesthesia, Faculty of Medicine, King Abdulaziz University, Jeddah, Saudi Arabia; 10 Department of Data Science, INTI International University, Nilai, Malaysia; 11 Department of Quality Enhancement Cell, Fatima Jinnah Medical University, Lahore, Pakistan; 12 Department of Radiology, University of Lahore, Lahore, Pakistan; 13 Department of Pathology, Faculty of Medicine, King Abdulaziz University, Rabigh, Saudi Arabia

**Keywords:** amitriptyline, chronic migraine, drug safety, headache impact test-6, migraine prophylaxis, topiramate

## Abstract

**Background:**

Chronic migraine is a chronic disabling neurological disease and has a significant impact on quality of life. Topiramate and amitriptyline are commonly used for migraine prevention, but treatment decisions should consider their effectiveness and safety profiles. This study aimed to compare the effectiveness and safety of topiramate and amitriptyline in chronic migraine.

**Methods:**

We conducted a quasi-experimental study at a specialised healthcare facility in Lahore, Pakistan, over a one-year period from March 2023 to March 2024. A total of 330 patients meeting the ICHD-3 criteria for chronic migraine were enrolled, including group (a) 165 patients treated with topiramate and group (b) 165 treated with amitriptyline. We used monthly headache frequency and the Headache Impact Test-6 (HIT-6) score to measure headache frequency and headache-related disability at baseline and at Weeks 4, 8, and 12. Adverse events were noted during the study period. We analysed the data using independent-samples t-tests, paired-samples t-tests, and the Chi-Square test, respectively.

**Results:**

The mean age of both groups (a) and (b) was 32.37 ± 8.48 years and 34.49 ± 9.02 years, respectively. Both treatments effectively decreased headache frequency and HIT-6 scores during the 12-week follow-up. Mean headache frequency was significantly reduced in group (a) vs. group (b) at week 12 (0.85 ± 0.63 vs. 1.22 ± 0.81 headache days per month, respectively, *p* < 0.001). Similarly, at Week 12, the mean HIT-6 score was significantly lower in the topiramate group than in the amitriptyline group (49.58 ± 6.16 vs. 51.64 ± 5.94, respectively; *p* = 0.002). Overall headache frequency decreased from 20.39 ± 4.23 episodes/month at baseline to 1.03 ± 0.75 episodes/month at week 12 (*p* < 0.001), while HIT-6 scores decreased from 68.14 ± 4.44 to 50.61 ± 6.13 (*p* < 0.001). Adverse events were reported in 25.5% of the patients taking topiramate and 35.8% of the patients taking amitriptyline (*p* = 0.042).

**Conclusion:**

Topiramate and amitriptyline both were effective in decreasing headache frequency and headache-related disability among chronic migraine patients. Topiramate was slightly more effective and better tolerated than amitriptyline.

## Introduction

Migraine is a prevalent, chronic, and disabling neurovascular disorder characterized by recurrent episodes of severe headache accompanied by autonomic symptoms, and in a subset of patients, transient neurological disturbances known as aura (1). Effective migraine management requires both prompt treatment of acute attacks and implementation of preventive strategies, including pharmacological and non-pharmacological interventions (2). The primary objectives of preventive therapy are to decrease the frequency and severity of migraine episodes, enhance responsiveness to acute treatments, improve daily functioning, and reduce migraine-related disability (3).

Despite extensive research, the precise mechanisms underlying migraine remain incompletely understood, resulting in the use of diverse pharmacological agents for prophylactic treatment. Advances in the understanding of migraine pathophysiology have shifted the conceptual framework of the disease from a predominantly vascular disorder to a complex neurovascular condition with significant central nervous system (CNS) involvement. Consequently, current preventive approaches increasingly focus on modulating neuronal excitability and neurotransmitter systems rather than solely targeting intracranial vascular tone (4). Within this context, antidepressants and antiepileptic drugs (AEDs) have emerged as important therapeutic options for migraine prevention.

Amitriptyline, a tricyclic antidepressant that inhibits the reuptake of serotonin and norepinephrine, has demonstrated well-established effectiveness in the management of chronic pain syndromes and migraine prophylaxis (5–7). In addition, it provides clinical value in patients with coexisting migraine and depressive symptoms (7, 8). Topiramate, a broad-spectrum antiepileptic agent with neuromodulatory properties, exerts its effects through multiple mechanisms, including modulation of voltage-gated sodium and calcium channels and inhibition of excitatory glutamatergic neurotransmission via non-N-methyl-D-aspartate (NMDA) receptors (8, 9). Clinical studies have established topiramate and amitriptyline as effective pharmacological options for migraine prevention (10)**.** Both agents have reduced migraine frequency and severity; however, differences in their mechanisms of action, adverse-effect profiles, and impact on quality of life may influence treatment selection in clinical practice. Comparative evidence evaluating their effectiveness and tolerability in patients with chronic migraine remains important for optimizing individualized treatment strategies. Therefore, the present study aims to compare the effectiveness and safety of topiramate and amitriptyline in the prophylactic management of chronic migraine by assessing headache frequency, headache-related disability, and treatment-associated adverse events over a 12-week follow-up period.

## Methods

A quasi-experimental study was conducted at specialized healthcare facility from March 2023 to March 2024, after obtaining approval from the Ethics Review Committee (IRB Ref. No. 365/Medicine/ICH). Sample size calculation was performed using the World Health Organization (WHO) sample size calculator based on headache frequency outcomes reported by Houts et al. (11) Assuming a mean headache frequency of 0.65 ± 0.81 days per month in the topiramate group and 0.91 ± 0.87 days per month in the amitriptyline group, a minimum sample size of 165 participants per group was required to achieve 80% statistical power at a 95% confidence level.

### Patients selection

The study included patients aged 18-55 who met the criteria of the International Classification of Headache Disorders, Third Edition (ICHD-3) for chronic migraine (CM), regardless of sex. The patients excluded from the study were patients with secondary headache related to trauma, tumors, stroke, infections or other neurological conditions, pregnant and lactating women, patients with severe hepatic, renal or cardiovascular disease, patients with known hypersensitivity to topiramate or amitriptyline, and patients receiving other migraine prophylactic medication.

Eligible patients were recruited using consecutive sampling after obtaining written informed consent. Demographic and clinical data such as age, sex, body mass index (BMI), duration of migraine, headache frequency, headache severity were collected on a structured proforma for patients who took topiramate as part of routine clinical care (Group a) and those who took amitriptyline (Group b). Information on medication-overuse headache, relevant medical and psychiatric comorbidities, and previous preventive-treatment exposure or failure was not systematically collected. Monthly headache frequency was defined as the number of calendar days during the preceding month on which a participant experienced a headache of any duration. Multiple headache attacks occurring on the same calendar day were counted as one headache day. Therefore, this outcome represents monthly headache days and does not distinguish migraine days from other headache days.

As treatment allocation was based on routine clinical decision-making rather than random assignment, the study employed a quasi-experimental design to compare outcomes between the two groups. The specific reasons for selecting topiramate or amitriptyline, including whether baseline BMI, anticipated weight-related effects, comorbidities, or patient preference influenced treatment choice, were not systematically recorded. The Headache Impact Test-6 (HIT-6) is a validated 6-item instrument to evaluate the impact of headache on social function, role function, vitality, cognitive function, and psychological distress, and was used to assess migraine-related disability. The total HIT-6 score ranges from 36 to 78; higher scores indicate greater headache-related disability and lower quality of life (12).

A total of 500 patients were screened for eligibility. Of these, 65 were excluded because they did not meet the inclusion criteria or declined participation. Among the 435 eligible patients, 105 were excluded before treatment initiation or final analysis because of treatment refusal, loss to follow-up, or incomplete baseline assessments. Consequently, 330 patients were enrolled and completed the study, with 165 patients allocated to each treatment group (Figure 1).

**Figure 1:**
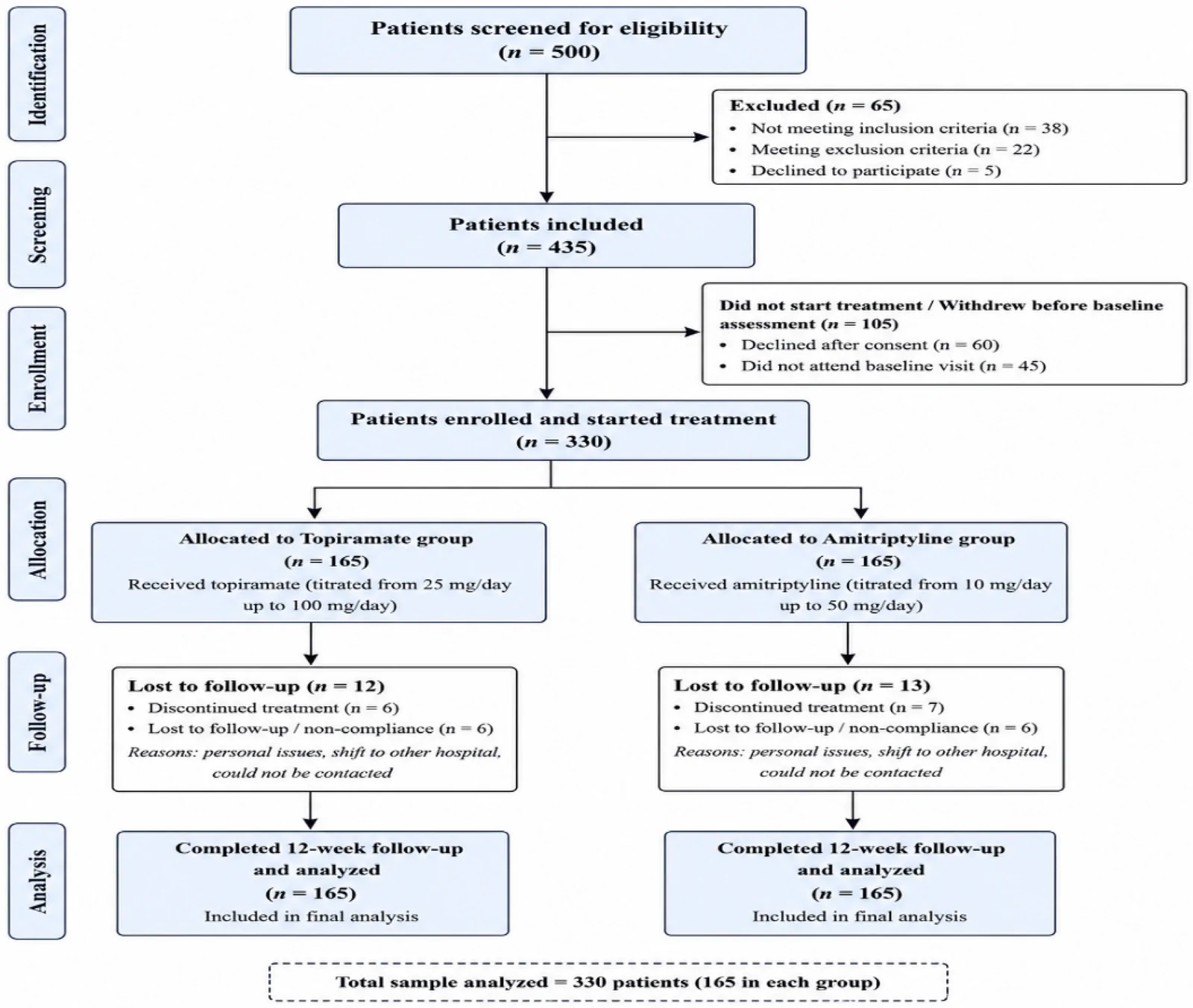
Flow diagram showing participant screening, enrollment, treatment allocation, follow-up, and final analysis in this comparative study evaluating.

### Management protocol

Patients in Group (a) were treated with topiramate, which was started at 25 mg/day and then titrated to 100 mg/day based on clinical response and tolerability. Patients in Group (b) were given amitriptyline, starting at 10 mg/day and increasing to a maximum of 50 mg/day as tolerated. Both groups received treatment for 12 weeks. Participants were permitted to use acute symptomatic medication when required as part of routine clinical care. No different rescue-medication protocol was applied between the treatment groups. However, the type, dose, and frequency of acute medication use were not systematically recorded. Follow-up assessments were performed at weeks 4, 8, and 12. To evaluate treatment effectiveness, the HIT-6 item was repeated at each follow-up visit. At each follow-up visit, participants were assessed for treatment-emergent adverse events. Reported adverse events, including paresthesia, hypoesthesia, nausea, sinusitis, xerostomia, fatigue, somnolence, and weight gain, were documented during follow-up. Any additional adverse events spontaneously reported by participants were also recorded. The observed frequencies and distributions of adverse events were reported. The main outcome was the difference between baseline and week 12 in the number of headaches and HIT-6 score. Secondary outcomes comprised treatment tolerability and adverse events.

### Statistical analysis

For quantitative variables, we used mean ± standard deviation, and for qualitative variables, frequencies and percentages. Continuous data were compared between the two groups using an independent-samples t-test, and repeated comparisons were performed at each predefined follow-up time point—the intra-group changes from baseline to week 12 using a paired-samples t-test. Chi-square test or Fisher's exact test was used to compare categorical variables. A *p*-value of ≤0.05 was considered statistically significant. The data were entered and analyzed using the Statistical Package for the Social Sciences (SPSS), version 26.0.

## Results

The mean age of patients receiving topiramate (group a) and amitriptyline (group b) was 32.37 ± 8.48 and 34.49 ± 9.02 years, respectively. Similarly, the mean BMI was significantly lower in the topiramate group than in the amitriptyline group (25.89 ± 3.53 vs. 27.25 ± 3.59 kg/m^2^, *p* = 0.001), representing an important baseline imbalance in this non-randomized study (Table 1). No statistically significant between-group differences were observed in sex distribution (*p* = 0.446), migraine duration (*p* = 0.517), baseline monthly headache days (*p* = 0.969), or baseline HIT-6 score (*p* = 0.520).

**Table 1:** Baseline demographic and clinical characteristics of study participants.

Variable	Topiramate (*n* = 165)	Amitriptyline (*n* = 165)	*p*-value
Age (years)	32.37 ± 8.48	34.49 ± 9.02	0.028
Male, *n* (%)	76 (46.1)	83 (50.3)	0.446
Female, *n* (%)	89 (53.9)	82 (49.7)	
BMI (kg/m^2^)	25.89 ± 3.53	27.25 ± 3.59	0.001
Duration of migraine (years)	5.12 ± 2.45	5.30 ± 2.64	0.517
Baseline headache frequency (episodes/month)	20.38 ± 4.18	20.40 ± 4.28	0.969
Baseline HIT-6 score	68.30 ± 4.60	67.98 ± 4.29	0.520

*BMI, body metabolic index; HIT, Headache Impact Test. Data presented as mean (SD, Standard Deviation).

### Effect of the treatment on the monthly headache days and HIT-6

Mean monthly headache days decreased progressively in both treatment groups during the 12-week follow-up period. No significance between-group difference was observed at Week 8 (55.17 ± 5.03 vs. 55.62 ± 5.46; *p* = 0.432), indicating a temporary convergence in HIT-6 scores before the difference re-emerged at Week 12 (Table 2 and Figure 2).

**Table 2:** Comparison of monthly headache frequency between groups during follow-up.

Time Point	Topiramate (*n* = 165)	Amitriptyline (*n* = 165)	*p*-value
Baseline	20.38 ± 4.18	20.40 ± 4.28	0.969
Week 4	11.42 ± 2.84	11.42 ± 3.01	0.978
Week 8	5.60 ± 1.76	6.16 ± 2.06	0.008
Week 12	0.85 ± 0.63	1.22 ± 0.81	<0.001

*Data presented as mean (SD, Standard Deviation).

**Figure 2:**
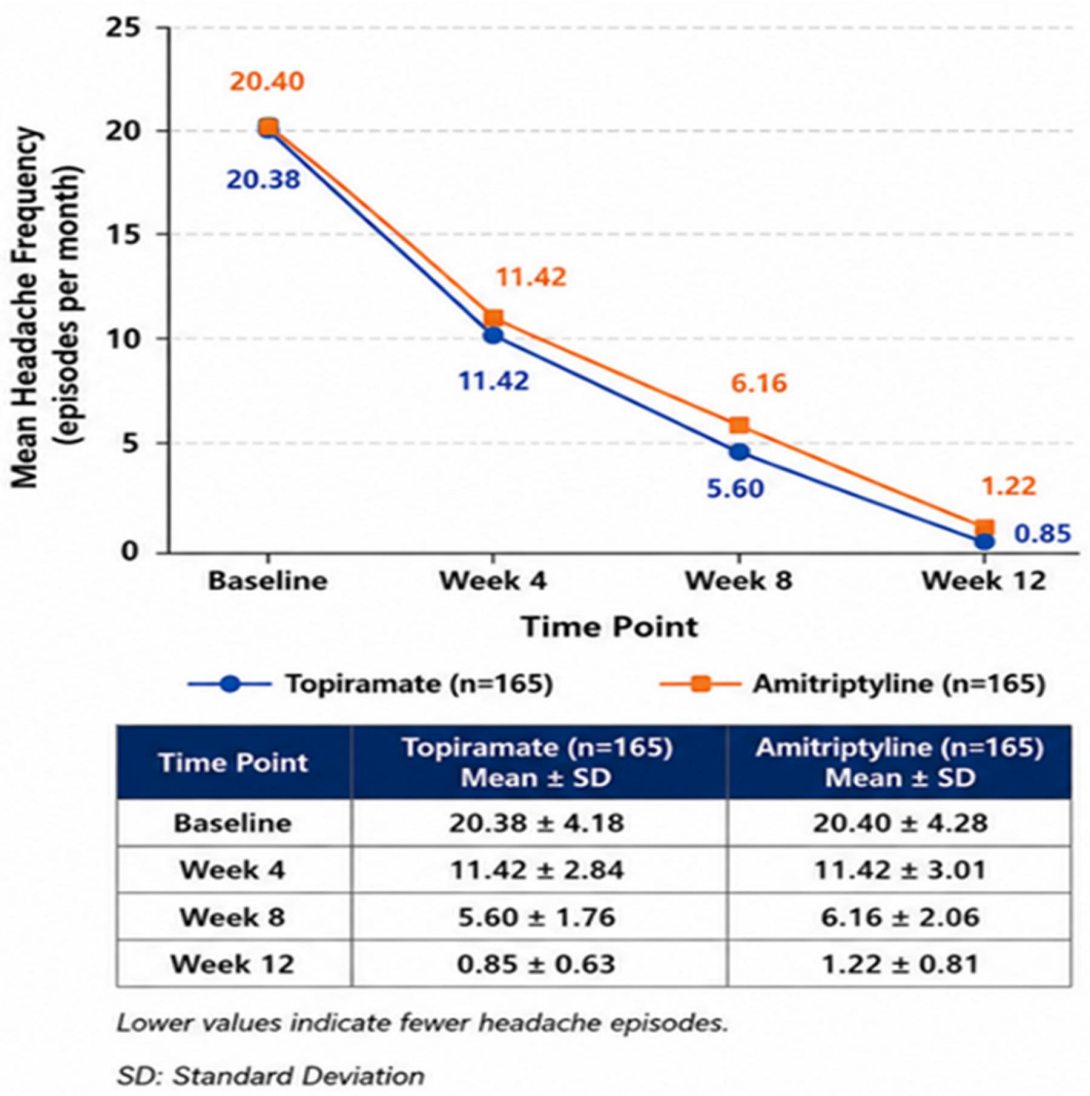
Trend in headache frequency over time.

At baseline, both groups had mean HIT-6 scores above 60, indicating a severe headache-related impact. Compared with the amitriptyline group, the topiramate group had a significantly lower mean HIT-6 score at Week 4 (60.47 ± 5.38 vs. 62.74 ± 5.32; *p* < 0.001) and Week 12 (49.58 ± 6.16 vs. 51.64 ± 5.94; *p* = 0.002). No significant between-group difference was observed at Week 8 (55.17 ± 5.03 vs. 55.62 ± 5.46; *p* = 0.432) (Table 3 and Figure 3).

**Table 3:** Comparison of HIT-6 scores between groups during follow-up.

Time Point	Topiramate (*n* = 165)	Amitriptyline (*n* = 165)	*p*-value
Baseline	68.30 ± 4.60	67.98 ± 4.29	0.520
Week 4	60.47 ± 5.38	62.74 ± 5.32	<0.001
Week 8	55.17 ± 5.03	55.62 ± 5.46	0.432
Week 12	49.58 ± 6.16	51.64 ± 5.94	0.002

*Data presented as mean (SD, Standard Deviation).

**Figure 3:**
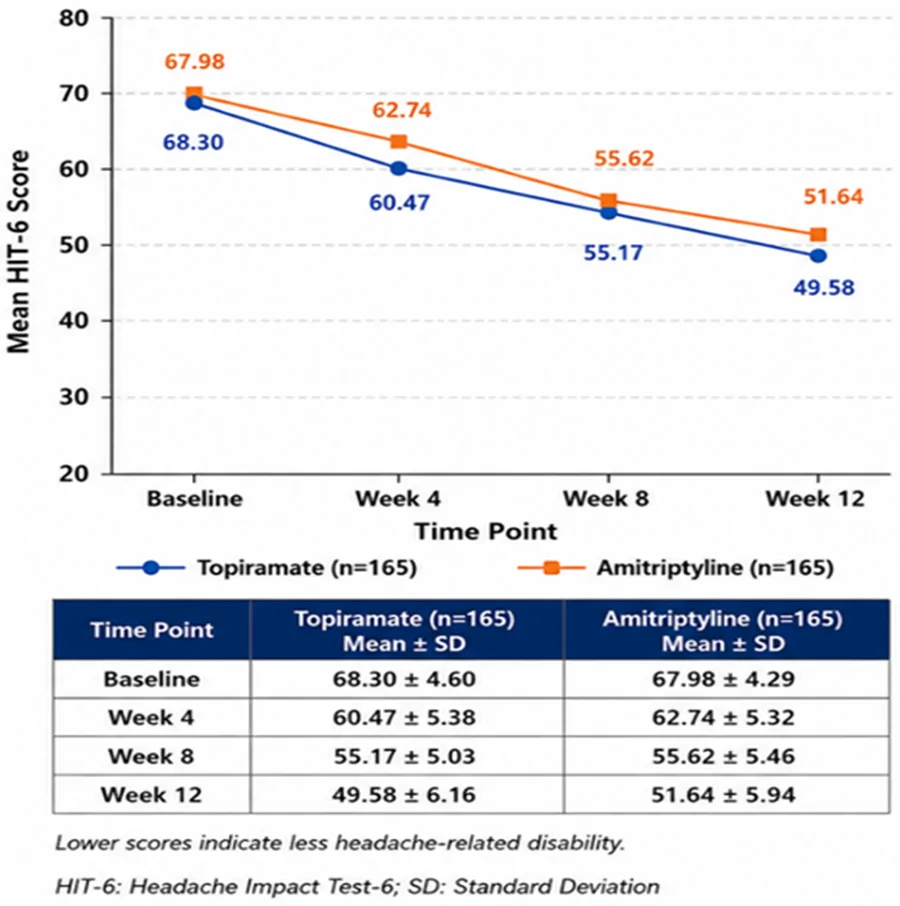
Trend in HIT-6 score over time.

In the overall study cohort, combining participants from both the topiramate and amitriptyline groups (*n* = 330), mean monthly headache days decreased from 20.39 ± 4.23 at baseline to 1.03 ± 0.75 at Week 12, corresponding to a mean reduction of 19.36 days per month (t = 83.06, *p* < 0.001). Similarly, the mean HIT-6 score decreased from 68.14 ± 4.44 at baseline to 50.61 ± 6.13 at Week 12, corresponding to a mean reduction of 17.53 points (95% CI: 16.72–18.34; t = 42.69; *p* < 0.001) (Table 4).

**Table 4:** Comparison of headache frequency and HIT-6 between baseline and week 12.

Outcome Variable	Baseline Mean ± SD	Week 12 Mean ± SD	Mean Difference	95% CI	t-value	*p*-value
Headache frequency (episodes/month)	20.39 ± 4.23	1.03 ± 0.75	19.36 ± 4.23	18.90–19.82	83.06	<0.001
HIT-6 score	68.14 ± 4.44	50.61 ± 6.13	17.53 ± 7.46	16.72–18.34	42.69	<0.001

*(Paired Sample t-test Analysis). Data presented as mean (SD, Standard Deviation).

### Adverse events and treatment tolerability

Adverse events were reported by 25.5% of patients in group (a) and 35.8% of those in group (b). The overall incidence of adverse events was significantly higher in the group (b) (*p* = 0.042), suggesting comparatively better tolerability of group (a) (Table 5). Neurological adverse events, including paresthesia, hypoesthesia, and nausea, were more frequently reported in the topiramate group, whereas xerostomia, somnolence, and weight gain were more common among patients receiving amitriptyline (Figure 4).

**Table 5:** Comparison of overall adverse events between groups.

Adverse Event	Topiramate n (%)	Amitriptyline n (%)	*p*-value
No	123 (74.5)	106 (64.2)	
Yes	42 (25.5)	59 (35.8)	0.042

**Figure 4:**
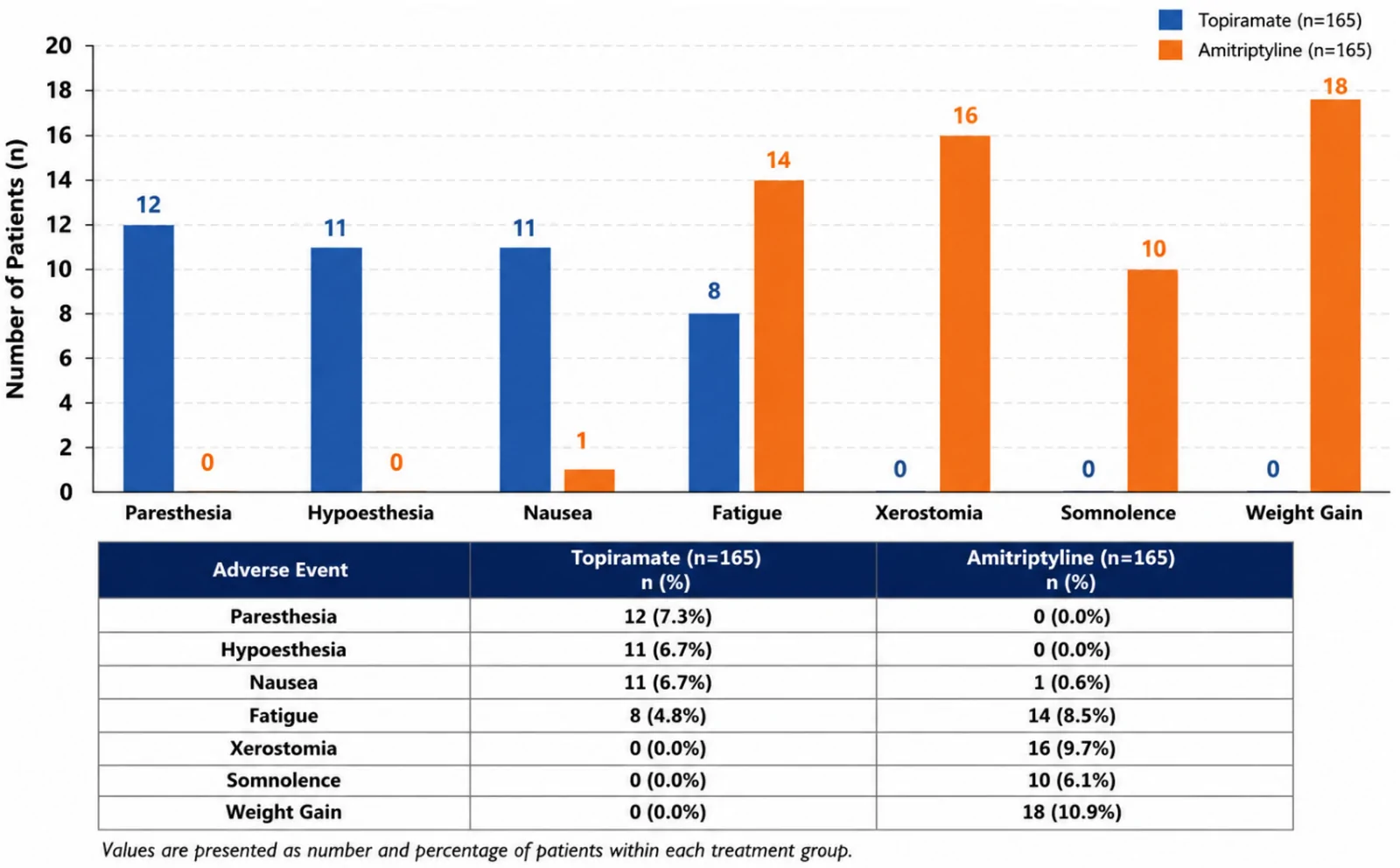
Adverse events by treatment group.

## Discussion

The current study evaluated the effectiveness and safety of topiramate and amitriptyline in the prophylactic treatment of chronic migraine. Both therapies produced substantial reductions in headache frequency and headache-related disability during the 12-week follow-up period. However, patients treated with topiramate in group (a) demonstrated significantly greater reductions in monthly headache frequency and HIT-6 scores compared with those receiving amitriptyline in group (b). Topiramate was also associated with a lower overall incidence of adverse events, suggesting a modest advantage in tolerability.

A principal finding of this study was the marked reduction in headache frequency observed in both treatment groups. This finding is clinically important because migraine remains one of the leading causes of neurological disability worldwide, and preventive therapy plays a critical role in reducing disease burden and improving quality of life (1–4). At baseline, patients experienced approximately 20 headache days per month, consistent with the substantial clinical burden associated with chronic migraine. Following 12 weeks of treatment, headache frequency decreased significantly in both groups, supporting the effectiveness of both topiramate and amitriptyline as preventive agents. These findings are consistent with previous evidence supporting amitriptyline as a preventive treatment for migraine (7, 10, 13–15) and with clinical trials and observational studies demonstrating the effectiveness of topiramate in migraine prevention (9, 10, 16–18)**.**

Although both medications were effective, topiramate produced a significantly greater reduction in headache frequency at weeks 8 and 12. By the end of follow-up, patients receiving topiramate reported less than one headache episode per month on average, compared with approximately 1.2 episodes per month among those receiving amitriptyline. Similar results were reported by Houts et al., who demonstrated that topiramate provided preventive effectiveness at least comparable to amitriptyline while maintaining a favorable overall therapeutic profile (11). Topiramate's superior performance may be attributable to its multimodal mechanism of action, including modulation of voltage-gated sodium and calcium channels, enhancement of inhibitory GABAergic neurotransmission, and suppression of excitatory glutamatergic signalling (8, 17–20). Through these mechanisms, topiramate may more effectively attenuate cortical hyper excitability and central sensitization, both of which are implicated in migraine pathophysiology. The present study also demonstrated substantial improvements in headache-related disability as measured by the HIT-6 questionnaire. Baseline HIT-6 scores exceeded 68 points in both groups, reflecting severe headache-related impairment. Significant reductions were observed throughout treatment, with the group (a) achieving lower disability scores than the group (b) at week 12. The absence of a significant between-group difference at Week 8 may represent temporary convergence in treatment response or normal variability during dose titration. However, because adherence, achieved dose, and acute medication use were not evaluated separately at each follow-up visit, this finding cannot be explained definitively. The observed improvements extended beyond reducing headache frequency and were accompanied by meaningful gains in daily functioning and quality of life. These results highlight the broader impact of preventive migraine therapy and support the inclusion of patient-reported outcomes when assessing treatment effectiveness (2, 12).

The effectiveness of amitriptyline observed in this study remains clinically relevant. Amitriptyline has been widely used for migraine prevention for several decades and continues to be recommended in clinical practice guidelines (3, 13–16). Its therapeutic effects are believed to result from modulation of serotonergic and noradrenergic pathways involved in pain transmission and central pain processing. In addition, amitriptyline may offer benefits for patients with coexisting depression, anxiety, insomnia, or chronic pain syndromes, conditions that frequently accompany migraine and contribute to disease burden (7, 21, 22). Therefore, despite the slightly superior effectiveness observed with topiramate, amitriptyline remains an important therapeutic option in appropriately selected patients.

Both medications demonstrated acceptable safety profiles, although adverse events were significantly more common among patients receiving amitriptyline. The observed adverse-event patterns reflected each drug's established pharmacological characteristics. Neurological symptoms, including paresthesia and hypoesthesia, were reported more frequently in the group (a), whereas xerostomia, somnolence, and weight gain predominated among group (b). These findings are comparable to those reported in previous studies evaluating migraine prophylaxis (9, 10, 17–20). The lower overall adverse-event burden associated with topiramate may contribute to improved treatment adherence, an important determinant of long-term therapeutic success. Clinically, treatment selection should remain individualized, taking into account effectiveness, tolerability, comorbidities, and patient preferences (2, 3, 16). While topiramate may be favored when maximizing headache control and minimizing adverse effects are priorities, amitriptyline may be particularly beneficial for patients with coexisting depression, sleep disturbances, or chronic pain conditions.

### Limitation

Several limitations should be considered. This single-center study may have limited generalizability. Treatment allocation was nonrandomized, creating risks of selection bias and residual confounding. The significant baseline BMI difference may reflect prescribing decisions based on anticipated weight-related effects; however, treatment-selection rationale was not recorded. Medication-overuse headache, medical and psychiatric comorbidities, previous preventive-treatment exposure or failure, and the type, dose, and frequency of acute symptomatic medication use were not systematically recorded. These omissions limited assessment of clinical representativeness and may have influenced monthly headache days and HIT-6 scores. Headache frequency was also based on participant-reported headache days and did not distinguish migraine days from other headache days. Therefore, the unusually large 12-week reduction in monthly headache days should be interpreted cautiously.

## Conclusion

Both topiramate and amitriptyline were effective preventive therapies for chronic migraine, producing significant reductions in headache frequency and headache-related disability over the 12-week treatment period. However, in the unadjusted between-group comparisons, topiramate was associated with fewer monthly headache days, lower HIT-6 scores, and a lower overall incidence of reported adverse events than amitriptyline. These findings emphasize that topiramate may offer a therapeutic advantage for some patients requiring long-term migraine prophylaxis. Further multicenter studies with longer follow-up periods are warranted to confirm these findings and evaluate long-term effectiveness and safety.

## Data Availability

The raw data supporting the conclusions of this article will be made available by the authors, without undue reservation.
